# Clinical performance of AI-integrated risk assessment pooling reveals cost savings even at high prevalence of COVID-19

**DOI:** 10.1038/s41598-024-59068-6

**Published:** 2024-04-17

**Authors:** Farzin Kamari, Esben Eller, Mathias Emil Bøgebjerg, Ignacio Martínez Capella, Borja Arroyo Galende, Tomas Korim, Pernille Øland, Martin Lysbjerg Borup, Anja Rådberg Frederiksen, Amir Ranjouriheravi, Ahmed Faris Al-Jwadi, Mostafa Mansour, Sara Hansen, Isabella Diethelm, Marta Burek, Federico Alvarez, Anders Glent Buch, Nima Mojtahedi, Richard Röttger, Eivind Antonsen Segtnan

**Affiliations:** 1 Department of Neurophysiology, Institute of Physiology, Eberhard Karls University of Tübingen, Tübingen, Germany; 2 Ellergy, Odense, Denmark; 3 Department of Mathematics and Computer Science, University of Southern Denmark, Odense, Denmark; 4 Innovation Unit, IdISSC, Hospital Clínico San Carlos, Madrid, Spain; 5 Grupo de Aplicación de Telecomunicaciones Visuales, Universidad Politécnica de Madrid, Madrid, Spain; 6 Easyrobot, Bratislava, Slovakia; 7 Hospital of Psychiatry, Odense, Denmark; 8 Research Center for Translational Medicine (KUTTAM), Graduate School of Sciences and Engineering, Koç University, Istanbul, Turkey; 9 School of Medicine, University of Southern Denmark, Odense, Denmark; 10 SDU Health Informatics and Technology, Maersk Mc-Kinney Moller Institute, Faculty of Engineering, University of Southern Denmark, Odense, Denmark; 11 Department of Engineering, Maersk Mc-Kinney Moller Institute, Faculty of Engineering, University of Southern Denmark, Odense, Denmark; 12 Department of Neurosurgery, Odense University Hospital, Odense, Denmark; 13 Synaptic ApS, Copenhagen, Denmark

**Keywords:** Scientific data, Microbiology

## Abstract

Individual testing of samples is time- and cost-intensive, particularly during an ongoing pandemic. Better practical alternatives to individual testing can significantly decrease the burden of disease on the healthcare system. Herein, we presented the clinical validation of Segtnan™ on 3929 patients. Segtnan™ is available as a mobile application entailing an AI-integrated personalized risk assessment approach with a novel data-driven equation for pooling of biological samples. The AI was selected from a comparison between 15 machine learning classifiers (highest accuracy = 80.14%) and a feed-forward neural network with an accuracy of 81.38% in predicting the rRT-PCR test results based on a designed survey with minimal clinical questions. Furthermore, we derived a novel pool-size equation from the pooling data of 54 published original studies. The results demonstrated testing capacity increase of 750%, 60%, and 5% at prevalence rates of 0.05%, 22%, and 50%, respectively. Compared to Dorfman’s method, our novel equation saved more tests significantly at high prevalence, i.e., 28% (*p* = 0.006), 40% (*p* = 0.00001), and 66% (*p* = 0.02). Lastly, we illustrated the feasibility of the Segtnan™ usage in clinically complex settings like emergency and psychiatric departments.

## Introduction

**Figure 1:**
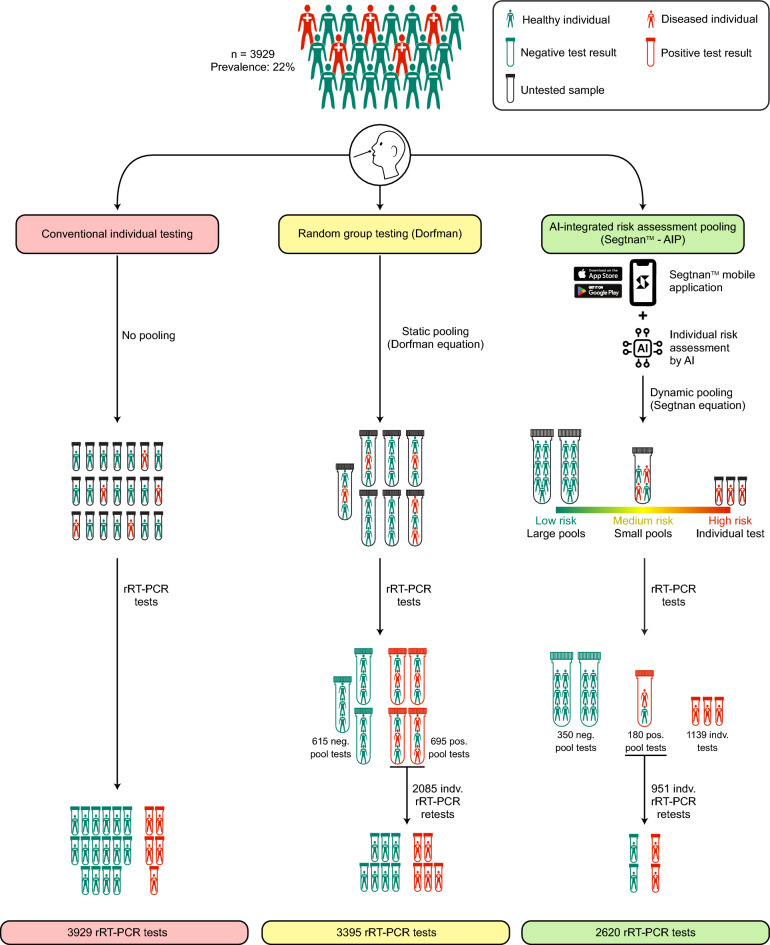
Schematic overview of the conventional individual testing (left), Dorfman random pooling (middle), and pooling based on the individual risk assessment and dynamic group sizes with the Segtnan equation using the mobile application (right). Number of tests are based on a disease prevalence of 22%.

Diagnostic biomarkers are conventionally tested individually with one test per sample. An alternative approach is group testing, where samples from various patients are pooled into groups^1–3^. This approach offers the advantage that if the test result of a whole pool is negative, all individuals in that pool can be ruled out, whereas a positive pool test outcome necessitates retesting of each pool members individually (Fig. 1).

The concept of group testing, initially introduced by Richard Dorfman^1^ during World War II, was revitalized during the COVID-19 pandemic. Its aim is to reduce the number of performed tests without compromising diagnostic performance. This reduction have been shown to lead to eightfold decrease in direct expenses^4,5^ or an exponentially increase in clinical test capacity^4^. The implementation of pooling for detection of SARS-CoV-2 is theoretically shown to increase test capacity by a factor of 18.6 (given a 2% prevalence)^6^, and practically reduce utilized tests by 58–83%^5,7^.

At present, there is no existing diagnostic gold-standard regarding sizes of pools in relation to the prevalence of the tested disease; the original Dorfman proposal utilized random pooling of samples, where group-sizes were based on the mathematical Dorfman’s equation (DE), which to date still is used as the mainstream for the determination of the optimal pool size^8^. The DE presents an inverse correlation between disease prevalence and pool size, starting with pools containing 11 samples at low prevalence (< 1%), gradually reducing the pool size to 3 samples above 15% prevalence. The inherent static nature of DE optimizes the economic benefits in pooling, regardless of the sensitivity and specificity of the test method. This means that the DE is financially unfeasible and not recommended at prevalence rates above 30%. Moreover, the differences in the point-prevalence of SARS-CoV-2 at a *local* test site^9–11^, dissimilar performance of the in-vitro diagnostic products, variation in lab equipment, and different national guidelines have all hampered a one-size-fits-all gold-standard, which would have optimize the diagnostic accuracy for both SARS-CoV-2 or any other pathogen^12^, that can be pooled. The applied optimal static pool size, for example, varies extensively in the literature, from 3 samples per group in Iran^7^, over 5 reported in Germany^13^, to 32–48 in Israeli studies^4,14^.

In 2021, “smart pooling” was proposed by Escobar et al*.* to overcome the problems of prevalence variability and failure in using group testing in settings with high prevalence^15^. Smart pooling uses a priori information about the patient to calculate the probability of an individual to be positive, based on the individual’s clinical information. Escobar et al*.* showed that in an area with a COVID-19 prevalence of 50%, smart pooling still could be 6% more efficient than conventional testing^15^. In another study, Deckerts et al*.* pooled individuals with the similar clinical background and found this more efficient compared to random pooling^4^. Despite these benefits, there still remain obstacles in data collection and processing for practical implementation in the clinical settings, hindering a widespread use of the concept. To the best of our knowledge, Escobar et al., is the only group that have implemented ML comparisons in the context of smart pooling and using real-world clinical data for this purpose. The research field is lacking any practical tool or product which can allow for day-to-day use of smart pooling in clinical routine.

In this study, we address the diagnostic challenges of pooling low and high prevalence rates by proposing personalized AI-integrated risk assessment pooling (AIP), integrated and individual accessible for patients through a mobile application, Segtnan™. AIP aims to enhance pooling’s diagnostic accuracy by applying generalized questionnaires, processed individually by an AI algorithm and a dynamic equation for pool sizes. The overall aim was to develop, improve and validate the diagnostic accuracy of the AIP, together with the clinical feasibility and applicability of the method. This was performed in 3,929 patients from Denmark and Spain, covering the clinical spectrum from entailed healthy individuals over inpatients at hospital wards to complex patients at somatic/psychiatric emergency rooms.

## Material and methods

### Data collection and preprocessing

**Figure 2:**
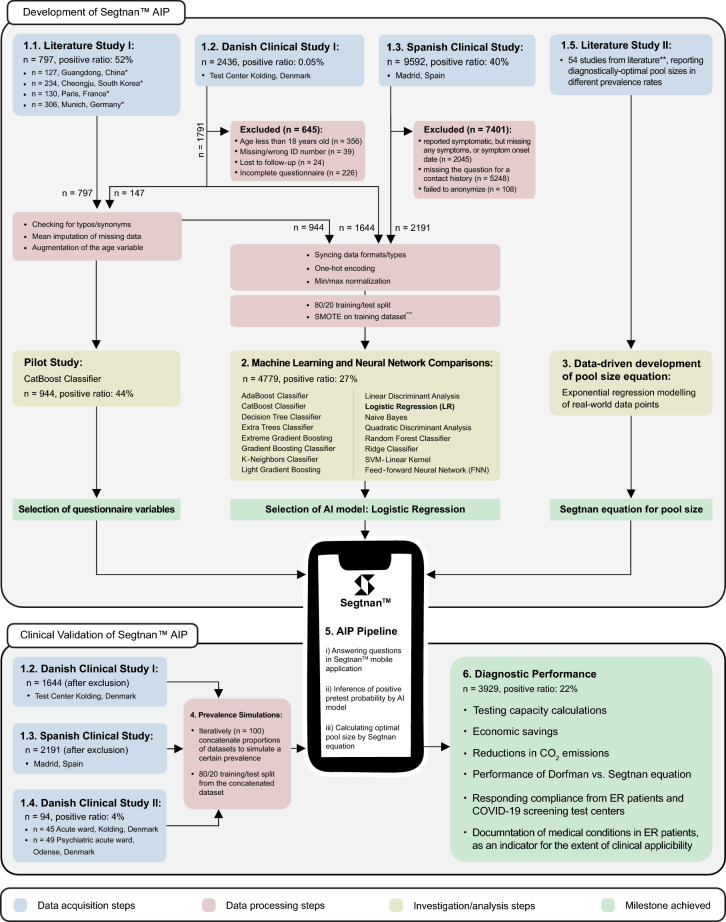
Flowchart of the study design. The figure illustrates the steps for the development of the Segtnan™ AIP pipeline (upper panel) and for the validation of the developed pipeline and the obtained results of its diagnostic performance (lower panel). The numbering of the colored rectangles indicates the respective section number in the Methods text. *, the data obtained from four literature studies^16–19^; **, reviewed in ref^8^; ***, for FNN the SMOTE step was replaced with balancing class weights^40^.

A flowchart of study design is presented in Fig. 2, illustrating the two interlinked study phases: the development of the Segtnan™ AIP (Fig. 2, upper panel) and its clinical validation (Fig. 2, lower panel). While the former may be of particular interest to the readers with a data science background, the latter is of special interest to the healthcare providers and clinicians. The data collection phase, illustrated in blue in Fig. 2, entails the datasets listed below. Following the description of datasets, the respective preprocessing steps are explained (also shown in red in Fig. 2).

#### Literature study I

**Figure 3:**
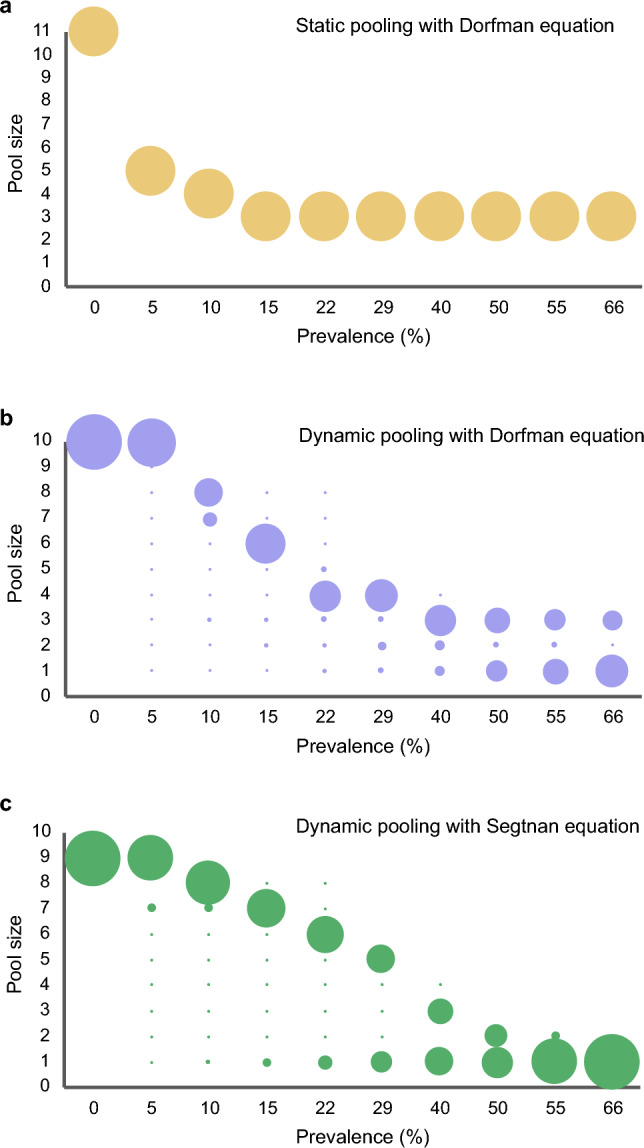
Group size using different pooling strategies in relation to prevalence. The original Dorfman equation with random group allocation of samples (**a**), individual risk assessment using AI-integrated risk assessment pooling (AIP) and group sizes according to the Dorfman equation (**b**) and using AIP together with the novel Segtnan equation (**c**).

Four early COVID-19 articles^16–19^, including n = 797 data entries, each corresponding to a real-time reverse transcription polymerase chain reaction (rRT-PCR) COVID-19 test result, obtained from 33 patients. Each data entry was evaluated to select relevant screening questions that were used in the clinical survey that was handed out to the patients in the succeeding prospective data collection phases (see 1.2–1.4 in Fig. 2). Each data entry contained the following: the real-time reverse transcription polymerase chain reaction (rRT-PCR) test result with the cycle threshold (CT) value (or the number of viral RNA copies per swab), the respective clinical symptoms of the patient on the day of sampling (and in some cases during the course of disease), contact history with a COVID-19 positive case, the dates of symptom onset and swab sampling, specimen used for sampling (e.g. nasopharyngeal, sputum, stool, etc.), the rRT-PCR gene targets, and the demographic information of the patient. Detailed elaboration on the extraction of the data entries is as follows:

i. Zou and colleagues presented 127 data entries from 17 patients in the study “SARS-CoV-2 Viral Load in Upper Respiratory Specimens of Infected Patients”^16^. The rRT-PCR CT values and the corresponding diagnoses were extracted from Fig. 1 of the cited article, and the clinical symptoms were available in the Supplementary Appendix from the same reference^16^. To note, each data point in Fig. 1 of the mentioned article is referring to one rRT-PCR test.
ii. Kim and colleagues presented 234 data entries from 2 patients in the study “Viral Load Kinetics of SARS-CoV-2 Infection in First Two Patients in Korea”^17^. The 234 data entries were extracted from Supplementary Tables 1 and 2 of the cited article^17^.
iii. Lescure and colleagues presented 130 data entries from 5 patients in the study “Clinical and virological data of the first cases of COVID-19 in Europe: a case series”^18^. The 130 data entries were extracted from Table 2 and Fig. 3, and the clinical course of the patients was extracted from the Tables S3-1 to S3-5 in the Supplementary Appendix of the cited article^18^.
iv. Wölfel and colleagues presented 306 data entries from 9 patients in the study “Virological assessment of hospitalized patients with COVID-2019”^19^. The 306 data entries were extracted from Fig. 2, and the clinical symptoms of patients were available in Table 2 of the cited article^19^.

The obtained raw data were further augmented in the below steps before the data were fed into the CatBoost machine learning (ML) algorithm^20^ (see left column in Fig. 2).

i. Typographical errors in symptom descriptions were manually corrected. The included symptoms were one or multiple of the following: recovered, low grade fever, high grade fever, chills, dry cough, productive cough, sore throat, malaise, myalgia, nasal discharge, tachypnea, dyspnea, hypoxemia, anosmia, diarrhea, nausea, headache, skin lesions, conjunctivitis, cyanosis, alteration of consciousness, other. In cases of fever with unknown temperature, the low-grade fever was selected. Fully synonymous medical terminologies were changed to the identical symptom from the list.
ii. Since many data entries were extracted from a few patients with certain age, without data augmentation, the trained model would develop an erroneous bias toward that specific age number. Hence, while maintaining the age category, random noise was applied on the age variable. This augmentation step was critical to obtain a generalized ML model.
iii. For data entries extracted from the fourth article^19^, the information about the gender and age were missing and could not be retrieved upon correspondence. These missing variables were imputed with mean imputation to keep the consistency of the dataset.

#### Danish clinical study I

Prospectively collected Danish patient data, n = 1791, collected at Kolding test center and Sygehus Lillebaelt, Kolding, Denmark with a point prevalence of 0.05%. Primarily, data from 2,436 patients were prospectively collected from the Hospital of Lillebælt and the Psychiatry Hospital, Odense, Denmark, February–March 2021. The inclusion criteria were individuals (symptomatic or asymptomatic) who were seeking a COVID-19 rRT-PCR test of inhabitants in the Region of Southern Denmark and signed the informed consent of participating in the clinical trial. From the included 2,436 individuals, 645 of them were excluded. The exclusion criteria (graphically displayed in Fig. 2) were age under 18 (n = 356); not having a Personal Danish ID number (n = 39); secondary refusal from any individual to be excluded from further analysis (n = 24); and an incomplete fill-out of the questionnaire (n = 226). After exclusion, the remaning 1791 individuals were registered with the following data: COVID-19 rRT-PCR diagnosis, demographic data, contact history, and the clinical symptoms and the medication history. The rRT-PCR tests were assessed in one of the four major laboratories: (a) Department of microbiology at the Hospital of Lillebælt, Vejle, Denmark; (b) Statens Serum Institute, Copenhagen, Denmark (SSI), (c) The Laboratory of Carelink, Kolding, Denmark (d) Department of microbiology, Odense University Hospital.

#### Spanish clinical study

Retrospectively collected Spanish patient data, n = 2191, collected at Hospital Clínico San Carlos in Madrid, Spain with a point prevalence of 40%. Data entries were collected from the available medical records at Hospital Clínico San Carlos, corresponding to patients with a COVID-19 rRT-PCR result performed during February 2020 and January 2021. The initial collection accounted for 9592 rRT-PCR tests belonging to 3700 patients. From these 9592 data entries, 7401 were excluded, leaving 2191 data entries belonging to 1084 patients. The exclusion criteria (graphically displayed in Fig. 2) were missing any symptoms after reporting to be symptomatic (n = 2045); lacking question of recent contact history (n = 5248); and failure to perform anonymization (n = 108). To comply with privacy regulations, the included dataset was fully anonymized, and the variables of age, date of symptom onset, and date of rRT-PCR sampling were generalized using k-anonymity^21^ (e.g., 28 years is represented as [25, 35) years) with k = 2. Those patients whose values could not be generalized were excluded (n = 108) to avoid identification. The reason for a high exclusion rate in the Spanish dataset was the retrospective design of the data collection, on the contrary to the Danish clinical studies.

#### Danish clinical study II

Prospectively collected Danish patient data from the emergency rooms (ER) of somatic (ER-S) (n = 45 with 0%-point prevalence) and psychiatric (ER-P) referrals (n = 49 with 4%-point prevalence) while investigating the patient compliance. This data was collected from the ER-S diseases at Sygehus Lillebælt (n = 45), and the ER-P at Psykiatrisygehuset (n = 49), Odense, Denmark. Age and gender variables were not collected in this data collection phase; however, the past medical history and the current diagnosis were actively documented (see Table 2).

All data from the above sources (1.1–1.4) were manually proofread to correct for any typos, out of range numbers, or wrong data format. In case of any discovered mistake, the data were retrieved again from the available medical documentations, if accessible. If such an access was not possible or the documentation was not complete, the data entry was discarded. In the Spanish dataset, for each data entry, a numeric age value was randomly selected from the age category that was assigned during the k-anonymity generalization. In all datasets, the categorical data (like symptoms and medications) were one-hot encoded, and all data were min/max normalized before ML and feedforward neural network (FNN) trainings. To correct for imbalances in dataset classes, the Synthetic Minority Over-sampling Technique^22^ (SMOTE) was used.

## Missing data

In order to use the datasets for ML and FNN training/testing, the missing values were handled as of the following: In the literature dataset (see left column in Fig. 2), the missing values were replaced by mean imputation within this dataset. In Danish and Spanish datasets (see middle column in Fig. 2), any entry containing a missing value was discarded, leaving only those that contained all variables (complete-case analysis). Regarding the ER datasets, they were not used for ML and FNN comparisons during the development of the Segtnan™ AIP (Fig. 2, upper panel) because the age and gender variables were missing in these datasets observing the respective privacy agreements at the time of data collection. However, during clinical validation (Fig. 2, lower panel), the ER datasets (Fig. 2; 1.4 Danish Clinical Study II) were included in the prevalence simulations, and the age and gender variables were randomly imputed from the distribution of the same variables in the Danish Clinical Study I. It is important to note that the medical conditions of patients upon arrival at the emergency room were only recorded in ER datasets (Table 2), and not in any other datasets. These medical conditions (past or recent medical history) were not used as a variable in AI investigations and were only recorded to illustrate the extent of patient compliance with the AIP questionnaire. We did not exclude any outliers from any datasets.

### Machine learning and neural network trainings

For the ML and NN trainings (see Fig. 2; 2 ML & FNN Comparisons), the following variables [variable types] were used: age [numeric], gender [dichotomous], subjective feeling of illness [in 6 ranks from 0 to 5], contact history [dichotomous], days on COVID-19-related medication [numeric], days since symptom onset [numeric], signs and symptoms [one-hot encoded], recent medication history [one-hot encoded], type of swab sampling [one-hot encoded]. The output variable was the result of the rRT-PCR test [dichotomous, i.e., two classes of positives and negatives]. The preprocessing steps contained one-hot encoding for categorical data and min/max normalization. The training and testing datasets were split with an 80/20 ratio (train/test) (see Fig. 2; 4 Prevalence Simulations) Since the prevalence of disease can be different from one region to another, we equalized the class sizes (0.5: 0.5) with SMOTE^22^ method to train the ML models without an intrinsic toward the majority class. The SMOTE was only applied to the training dataset after the train/test split. The analysis without the SMOTE step is also available in the Supplementary Material.

Fifteen classification ML algorithms and an FNN were fitted to the data for comparison (see Supplementary Material for details) with the following evaluation metrics: cross-validation accuracy (Accuracy), Receiver Operating Characteristics—Area Under the Curve (AUC-ROC), Precision-Recall Curve—Area Under the Curve (AUC-PR), F1 score, sensitivity (Recall) and specificity (Precision). For ML trainings, we performed a stratified tenfold cross-validation and the reported evaluation metrics in the manuscript are those of cross validation, unless specified otherwise. We intentionally kept the patients who had multiple tests within the datasets, so that the trained models can also learn the disease probabilities during the course of the disease, given the days from symptom onset and the fluctuating symptoms variables. For the analysis with only one test from each patient, please see the Supplementary Material. The Python package PyCaret^23,24^ (https://pycaret.org/) was used as a low-code open source opportunity for rapid ML implementations. For the investigation of a feedforward neural network (FNN) model, the AutoKeras library was used.

### Data-driven equation for pool size

After the pretest probability calculation by an ML algorithm, we further investigated two approaches to calculate the optimal diagnostic pool size for each individual: (i) the original Dorfman equation (DE), and (ii) a proposed data-driven pool size equation named Segtnan Equation (SE). To derive the SE, we used the included articles reviewed in a study from 2021 by Evangeline et al*.*^8^ (1.5 Literature study II in Fig. 2), in which the results of the most optimal pool size for any given prevalence is systematically gathered (see the original Table 1 in ref^8^). We performed an exponential regression on this experimented real-world clinical data to obtain the SE (Fig. 2; 3 Data-driven development). The SE differs from the DE, which is focused on the economic optimum. In contrast, SE is based on results of studies that have already successfully implemented group testing and are reporting the diagnostic optimal pool size from real life data. That means SE provides diagnostically sensible outcomes, rather than only optimizing economic savings. The SE is written as:

$$m = \left\lfloor {9.7 \times e^{ - 0.06 p} } \right\rfloor$$

**Table 1:** The simulations of various prevalence rates were performed by iteratively combining certain proportions of Danish and Spanish datasets.

Intended prevalence	Simulated prevalence	Danish dataset (1.2)	Spanish dataset (1.3)
0.05%	0.05%	100%	–
5%	5.2%	100%	12.5%
10%	9.85%	100%	25%
15%	14.96%	100%	50%
20%	22%	100%	100%
30%	28%	50%	100%
40%	40%	–	100%
50%	50%	–	100% of Spanish dataset with 445 negative patients randomly removed
55%	55%	–	100% of Spanish dataset with 600 negative patients randomly removed
65%	66%		100% of Spanish dataset with 875 negative patients randomly removed

In which $p$ refers to the calculated positive pretest probability, $e$ is the Euler’s number, $m$ is the proposed group size, and ⌊⌋ is the mathematical notation for the floor function, i.e., ⌊*x*⌋ is the largest integer less than or equal to *x*. We first compared the original DE with population prevalence (i.e., random pooling) against the SE with individualized *a-priori* clinical information. Furthermore, we compared the DE against the SE considering the individualized *a-priori* for both. The substitution of the population prevalence with the calculated predicted positive probability in the DE is known as smart pooling^15^.

### Prevalence simulations

We compared DE with SE pool size approaches at various prevalence rates from 0.05 to 55% using concatenated new datasets. The intended prevalence rates were simulated by iteratively (n = 100) random sample a certain proportion of the original Danish (see 1.2 in Fig. 2, prevalence 0.05%) and Spanish (see 1.3 in Fig. 2, prevalence 40.1%) datasets, and further concatenating data subsets together. Table 1 demonstrates the proportions from the two datasets (1.2 and 1.3 in Fig. 2) to obtain the intended prevalence rate. The concatenated datasets with intended prevalence rates were randomly split in 80/20 train/test. Per each iteration, a Logistic Regression (LR) classifier model was further fit to the training dataset (80%) and tested on the unseen testing dataset (20%). The inference of the LR, i.e. the positive pretest probability, was fed into the pool size equation to determine the group size. Thereupon, the diagnostic performance outcomes of the simulation were calculated. The reported outcomes are the average of the 100 iterations, performed to minimize any random effects and sampling bias. As shown in Table 1, the obtained prevalence rates were trivially different from the intended prevalence rates, which is due to the abovementioned iterative random sampling from datasets.

### AIP pipeline

The AIP diagnostic tool is available as a commercially available mobile application, Segtnan™, freely downloadable for IOS and Android smart phones. The AIP pipeline can be summarized in three steps (see Fig. 2; 5 AIP Pipeline): (i) The patient fills in a clinically validated questionnaire in Segtnan™ mobile app. (ii) The answers are analyzed by an ML model and an individual *a-priori* likelihood per patient for being infected, hence the term AI-integrated risk assessment, is obtained. (iii) The *a-priori* likelihood is further used in the SE which proposes the optimal diagnostic size of the pool for the individual being tested. Lastly, the implementing laboratory pools together the samples with the same pool size number obtained from the mobile application. Individuals with identical pool numbers are not subjected to any additional algorithm for selection or prioritization when they are assigned to a pool. In other words, individuals of same pool size are simply pooled together until the pool size number is reached. The pool is tested when the number of its members reaches the calculated pool size (e.g., 3 individuals with a pool size of 3). Obviously, the laboratory may decide to test an immature pool, i.e., having less members than proposed pool size, based on its logistics and patient turnover. Implementing AIP does not imply pooling in all instances—if the positive pretest probability for the disease surpasses a diagnostic threshold determined by SE, the pool size will be one, resulting in individual testing. The pool size number is displayed as a number and a QR code.

### Outcome calculations

The mathematical estimation methods used for the analysis of AIP performance under various prevalence in terms of changes in testing capacity, economic savings and savings in CO_2_ emissions are comprehensively explained in Supplementary Material. Differences between used tests when applying (a) Dorfman pooling, (b) AIP + Dorfman Equation, or (c) AIP + Segtnan Equation were calculated using two-sample test of proportions and all calculations were performed using Stata 11 with an applied significance level of 0.05.

### Privacy and ethical considerations

The Segtnan™ AIP technology is made for heightening global diagnostic accuracy, preventing a potential new lockdown from Covid-X and ensuring maximal digital patient safety. When it comes to privacy, Segtnan™ mobile application is designed to avoid the use of personally identifiable information and to avoid any interaction with local hospital journals or healthcare IT-systems. With this architecture, the Segtnan™ can be used for worldwide scalability and comparison of scientific data across national borders. The Segtnan™ app uses HTTPS encryption protocol for all data transfers. It also creates Unique Universal Identifiers (UUID) which is used instead of national personal ID-numbers. It does not process, collect, or store the IP address or any equivalent information that could identify the end user’s device. Also, it does not store any cookies. The Segtnan™ end-users can only choose from pre-determined responses which do not allow for the provision of identifiable information. The responses are based on categories which encompass larger groups of persons such as subjective feeling of illness, a recent contact history, or general symptoms. The Segtnan™ is assessed to be outside the Medical Device Regulatory (MDR). The newest version of the privacy policy can be reached at https://www.segtnan.ai/privacy.

The study was approved on 19th of January 2021, by the regional Ethical Committee of Southern Denmark (Approval ID: 2021200-1) in the Region of Southern Denmark. All individuals participating were instructed about the study orally and in writing and participated voluntarily. Written informed consent was obtained from all participating subjects. All data were anonymized, and confidentiality reserved with limited access of data analysts. The study fully obeys with the seventh revision of Declaration of Helsinki, 2013. Authorship responsibilities have been consistent with authorship guidelines published by the International Committee of Medical Journal Editors (ICMJE).

## Results

### Patient data demographics

For the three original datasets (see “Methods” “Danish clinical study I”, “Spanish clinical study”, and “Danish clinical study II” Sections), which were originally collected during this study, characteristics of included patients (age, sex, COVID-19 symptoms at time of testing) and the underlying diseases are presented in Table 2. For a narrative description of datasets, see Supplementary Material.

**Table 2:** Patient characteristics and their medical conditions upon arrival at the emergency room. ER, emergency rooms.

Dataset variables	Danish dataset (1.2)	Spanish dataset (1.3)	ER datasets (1.4)
N	1791	2191	94
Mean Age (+/− variation)	44.7 y [30.2—59.2]	61.2 y [43.5—78.9]	–
Sex ratio	f:m = 1.17:1	f:m = 1.06:1	–
No symptoms at sampling	1675	1185	72
Symptomatic at sampling	116	1006	22
Schizophrenia/Schizoid Affective disorder	–	–	30
Bipolar	–	–	8
Depression	–	–	5
PTSD	–	–	3
Substance Abuse	–	–	6
Other	–	–	1
Cardiovascular disorders	–	–	25
Endocrinological disorders	–	–	28
Gastrointestinal tract disorders	–	–	14
Lung disease	–	–	7
Neurological disorders	–	–	7
Urological disorders	–	–	6
Kidney disease	–	–	4
Rheumatological disorders	–	–	4

### Machine learning results in AI-integrated risk assessment

We compared 15 machine learning algorithms (see Table S1 of Supplementary Material), in terms of how well they can correctly predict/classify the unseen data. We found that the ensemble and gradient boosting model categories, like CatBoost, light gradient boosting machine (LightGBM), and extreme gradient boosting (XGBoost), can predict the clinical diagnosis with the highest accuracy. CatBoost had the best accuracy (80.14%), followed by LightGBM (80.09%), and XGBoost (79.54%). The machine learning algorithms were mainly comparable in terms of accuracy ranging from 80.1 to 78.5% (excluding the quadratic discriminant analysis and the Naive Bayes classifier). We obtained an accuracy of 81.38% from the feed-forward neural network (FFN) Keras model which was superior to the ML models.

During the development of the AIP, we aimed to minimize the false negatives (i.e., high sensitivity). Specificity, however, was of less importance due to the guaranteed gold-standard detection of false positives during the step of individual retests (i.e., after a pool became positive). Hence, we searched for ML algorithms that provided a favorable precision-recall trade-off with a bias over recall. Considering among the ML models with reasonable accuracy, the support vector machine (SVM) had the highest recall of 84.6% followed by ridge classifier, linear discriminant analysis (LDA), and Logistic Regression (LR). The ROC-AUC was highest for gradient boosting classifier (ROC-AUC = 0.868, F1 = 68.0%), CatBoost (ROC-AUC = 0.864, F1 = 67.1%), Ada Boost Classifier (ROC-AUC = 0.859, F1 = 67.8%), and LR (ROC-AUC = 0.853, F1 = 67.7%). Based on the metrics presented, we selected LR as the ML of the AIP clinical tool (see Discussion for more). In the Supplementary Material, we have also provided the training details of the MLs and FNN.

### Prevalence and retesting of pools

**Figure 4:**
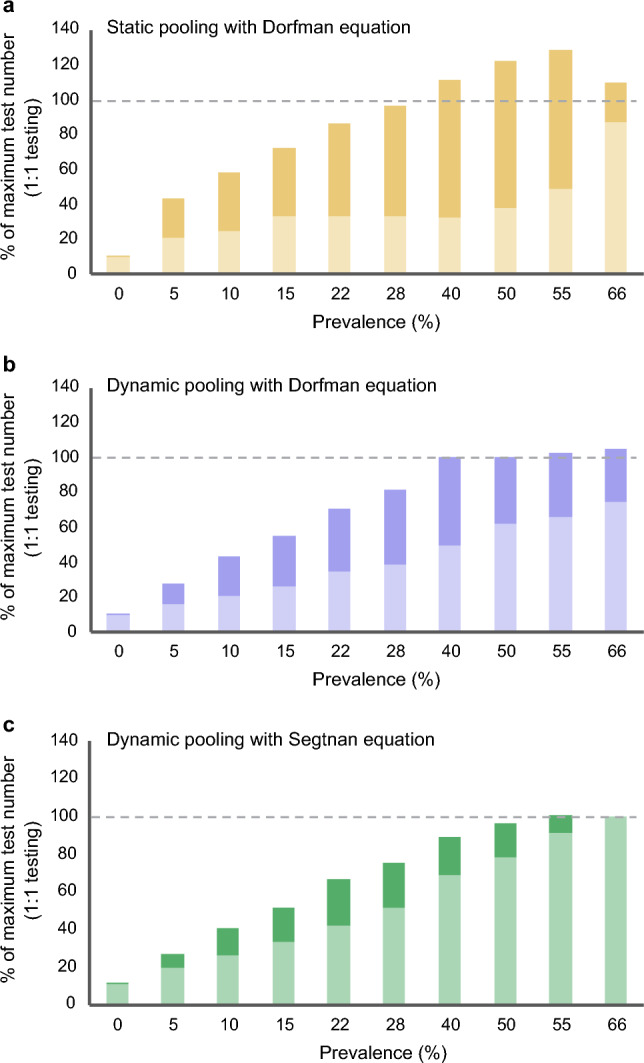
Testing efficacy as number of tests needed compared to the individual 1:1 testing (100% marked with a dashed line) for pooled test analyses (light color) + individual retesting (darker color). Data are presented for random pooling + Dorfman equation (**a**), AI-integrated risk assessment pooling (AIP) + Dorfman equation (**b**) and AIP + Segtnan equation (**c**).

Optimal pool sizes were estimated either using the DE on population prevalence (Figs. 3a and 4a) or individual risk assessment from the AIP algorithm, i.e., an individualized probability of a positive diagnosis (e.g., 30%) calculated per patient before pooling. The latter was compared using the DE (Figs. 3b and 4b) or using the novel SE (Figs. 3c and 4c). The results showed that the use of AIP decreased the numbers of tests that were retested, and it showed that the SE provided higher diagnostic accuracy in determining correct pool sizes in relation to prevalence, for healthy/non-healthy individuals, which in turn led to fewer retests all together in simulations with prevalence rates ranging from 0.05 to 50%. The theoretical calculation of utensils saved with the use of AIP + SE compared to 1:1 testing showed a reduction of 1.8 kg of CO_2_ emissions per 100 samples, see Supplementary Material for details.

### Testing capacity

The following three pooling strategies were tested: (a) “DE”, (b) “AIP + DE”, and (c) “AIP + SE”. We compared these strategies head-to-head with the conventional 1:1 test regime as the gold standard. Figure 4 depicts the relative usage of medical tests displayed in percentages compared to test regimes as we know it today (1:1 testing), divided into the number of tests performed as pooled tests and eventual individual retesting. Test requirement increased with population prevalence, from 12 tests (compared to 100 tests by individual testing) at a prevalence of 0.05% toward 96 tests at a prevalence of 50%. Above 50%, no added value was seen. Except for very low prevalence rates, the SE resulted in a reduced number of required tests than the DE (in average 5.8 less tests consumed per 100 tested samples). Statistically, the SE performed significantly better than the DE (using fewer tests) at prevalence rates of 28% (*p* = 0.006), 40% (*p* = 0.00001) and 66% (*p* = 0.02).

### Financial benefits

**Figure 5:**
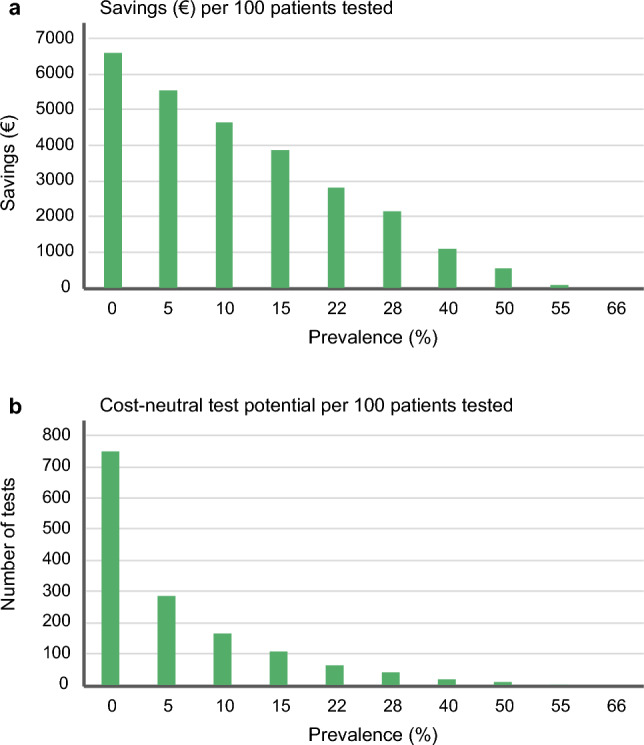
Financial benefit of AI-integrated risk assessment pooling compared to the conventional individual testing in relation to prevalence presented as savings in € per 100 tested samples (**a**) and the cost-neutral testing capacity (**b**).

Total cost estimations for testing procedure are complex involving many variables. An estimated price in Denmark per one hundred rRT-PCR samples would cost around 7500 €, based on a test price of 75 € per sample (see Supplementary Material) whereas retesting is estimated to be cheaper (64 €) due to the already-performed sample extraction and sample handling steps. Figure 5a shows the expected savings per 100 samples after applying the Segtnan™ AIP, starting from 6600 € at the lowest prevalence and decreasing as the prevalence rise. At prevalence rates above 50% financial benefits were reduced to less than 5 € per test on average. Despite the number of tests mimicking the 1:1 testing approach, there is a clear relation between the population prevalence and economic savings up to the prevalence rates of 55% when AIP + SE is used. Figure 5b addresses the amount of additional samples that can be tested as group-tests within the same cost as individual testing. At a low prevalence (0.05%), an increase of 750% in the test capacity was possible using AIP, prior the total cost exceeding the cost of traditional individual testing. With a prevalence of 15%, the total number of tests could be doubled compared to the conventional testing regime and with a prevalence at 22%, applying AIP could increase testing capacity with 60%.

### Validation in clinical settings

To investigate the feasibility of AIP in a clinical setting, we handed out our clinical questionnaire in various hospital wards and at a community test center that performed large scale routine screening during the lockdown. The questions were answered by patients either laying on their hospital beds in multi-bed or fully isolated wards, waiting rooms at ER wards, or standing in long lines at test centers. In a qualitative comparison, we found no difference between the three settings. Complicated somatic and psychiatric patients, as well as citizens with or without disease symptoms were able to cope with the questionnaire. Due to the qualitative nature, no data on response rate, nor unwillingness to participate were recorded.

## Discussion

This study showed that the AI-integrated risk assessment pooling (AIP) method significantly increases the test capacity by reducing the numbers and sizes of pools that needs individual retesting, compared to the conventional Dorfman’s pooling. We found AIP was additionally cost-effective for pooling at prevalence rates up to 50% and applying a novel data-driven pool-size equation (SE) produced fewer retests compared to the original economic-driven Dorfman equation. The method integrated into the mobile application Segtnan™ makes “individualized pooling” clinically feasible to perform in day-to-day clinical settings, even in patients with multiple complex somatic and psychiatric comorbidities.

### Prevalence and dynamic pool sizes

The AIP enables individual screening and dynamic pool size in correlation with local prevalence rates, in line with recommendation from recent articles on pooling^8,25^. We tested the AIP in disease-prevalence spectrum from 0.05% to 50%, similar to prevalence rates presented in recent comprehensive review ^8^. Here they compiling studies that have applied group testing successfully at low (0.2%^26^), moderate (5–10%^9,27^) and high prevalence rates (30–50%^15,25^). According to Eberhardt et al*.*^25^, pooling at low prevalence rates are diagnostically accurate and safe, while at higher prevalence rates, smaller pool sizes with lesser number of samples are to be chosen. They concluded that prevalence < 30% were beneficial, however there is no group testing scheme that seem optimal for all prevalence rates. This upper efficacy limitation above 30% prevalence is illustrated in Fig. 3a, where random pooling using DE exceeds the number of individual tests. In another study on 3303 positive samples and associated viral loads, it was shown that group testing with a dynamic pool size performs better concerning both sensitivity and test efficiency, compared to having a fixed pool size of 10^28^. Together, all this support our results; the optimal approach builds on dynamic and individualized pooling, as facilitated in the Segtnan™ mobile application.

We further found that the performance superiority of our clinically derived equation (SE) compared to the financially anchored DE could partly be due to smaller pools containing more homogenous members as prevalence increases, in contrast to Dorfman’s larger and random assigned pools (Fig. 3). This was additionally emphasized by the fraction of re-testing (Fig. 4), which was greater with DE than SE, again driven by too large pools. With such enhancements to the selection of the pool size and the ability to harvest individual data directly from the patient, the use of Segtnan™ AIP will ensure considerable benefits under future pandemics, even at prevalence rates up to 50%. Escobar et al*.* have also reported savings in high prevalence rates up to 50%^15^ which is aligned with our findings.

### Machine learning in AI-integrated risk assessment pooling

Our results proved that patients could answer as little as seven simple questions, and this is sufficient to calculate an individualized risk and diagnostically optimal pool size assessed by the Segtnan™ AIP. Certain questions were found to be less significant (left column in Fig. 2), by feature importance analysis, for the calculation of the pretest probability which is in line with the previous results from literature. Escobar et al*.*^15^ showed that from the seven categories of their collected variables, clinical signs and symptoms were more determinant for ML inference, compared to less descriptive background variables, like information upon family, healthcare or social status.

Our results showed that for the classification between disease and non-disease, the validation accuracy of CatBoost was maximum. However, we selected the LR as the optimal ML model for the Segtnan™, which was based on: (1) for product implementation, to increase the clinical sensitivity and minimize false negatives, we prioritized ML models with high sensitivity and AUC. This ensures a bias towards positive diagnoses so that no patients are overlooked. (2) in terms of validation accuracy, ML algorithms exhibited comparable performance, with LR displaying slightly lower accuracy compared to the CatBoost. (3) LR emerged as the most suitable choice due to its simplicity as a ML algorithm and the ease of monitoring its behavior in production. Such simplicity eliminates complexities in interpretability and enhances the comprehensibility of the results. Additionally, this is of special importance on a global scale implementation as it minimizes vulnerabilities to data poisoning attacks. (4) the file size (in terms of bytes) of the LR model is significantly smaller than its counterparts, facilitating the provision of historical records and archiving of models as an additional service for users. (5) the training and inference processes of the LR model require fewer computational resources, enabling easier scalability and even seamless implementation on mobile devices such as smartphones and tablets. This feature also contributes to the development of technical infrastructures where patient data can remain within the cell phone, ensuring data privacy and security.

Additionally, the feed-forward neural network (FNN) would need an initial ~ 3000 data entry to obtain a comparable accuracy to ML, a test size unfeasible for individual laboratories to collect, in case they want to train their own models. The training and inference time for the FNN was also considerably higher than any ML model. In other words, the implementation of FNN will require more advanced computational and processing units. When the patient data is meant to securely stay on the mobile device, such a computational-intensive task might not be possible, even at the inference level. With the selection of LR, in parallel to its high diagnostic accuracy, we have enabled the infrastructure for data security and global scalability.

### Strengths and weaknesses

A main strength of this study is the utilization of minimal information (i.e., as little data as possible), in contrast to *big data* approaches that might be not optimal for clinical settings. The minimal data context with the choose of a monitorable simple ML model like LR allows us to feasibly control for the training qualities and guarantee a safe, sustainable and functional platform (e.g., from false data injection attacks). Another strength is the implemented high privacy standards, meaning that the application does not ask for or collect any personal identification number, personal address, or any individual-specific identifiers. Further, the app is built on Flutter (Google, Mountain View, California, USA) with a particular emphasis upon the user privacy evident by its avoidance of collecting any IP addresses or utilizing any cookies. Using the Segtnan™ app guarantees a safe, feasible, and high-quality data collection process, built upon the standardized data collection protocol established during this study. Within the app’s user interface, critical questions cannot be left unanswered ensuring no missing variables during the data collection. Also, the collected data from the developed user interface are error-free in terms of data formatting, out of range numbers, inappropriate characters or typographical mistakes. Ensuring the absence of such errors or missing values is of course especially crucial for a feasible and scalable handling of the data with AI.

Furthermore, the generically programmed pipeline of the Segtnan™ AIP is ready to be clinically validated and used for pooling in other pathogens, e.g. HIV^2^, Chlamydia^29^, Malaria^30^, and Tuberculosis^31^. However, the data-driven pool size equations for these diseases (similar to SE) are yet open to be investigated. Other non-communicable diseases with non-parametric diagnostic indicator of Network Diaschisis for Alzheimer’s disease^32^, Parkinson^33^, stroke^34^, and glioma^35^ could potentially also benefit from the AIP method.

A general challenge of app-based approaches in healthcare is penetration of the intended segment, i.e., how many patients will use the solution. Mobile technology and smartphones are the most widespread communication form, also in developing countries^36^. Relying on app-based mobile communication is therefore considered an advanced, secure, and sustainable solution. Alternatively, if needed, local terminals at sample or test sites can be included; however, most potential patients, even in developing countries, will have access to an app-based solution. The driving force behind upscaling in all settings, both in highly developed western and undeveloped countries, is the patient—health care provider (HCP)—laboratory interaction, where patient’s participation should be motivated, recommended and/or referred by HCPs/laboratories, without being hampered by a time-consuming and complicated technical solution. We have shown in this study that even in complicated patients’ cases, which in many other settings avoid third-party interactions, can cope with the AIP questions without any issues, which is considerably important for the implementation in real clinical settings. In general, we have not documented any limitation regarding usability and patient heterogeneity among the complex group with multiple somatic and psychiatric comorbidity.

Another limitation of the current study was that it was not designed to investigate the response rate and satisfaction of the patients that provided data to our investigation. This may have led to a risk for selection bias in the data, especially in the case of ER-P and ER-S patients. Lastly, certain limitations did not allow for the current study to include a cohort design, in which the pooled tests and individual retests are performed prospectively in the laboratory. It is recommended that future studies use a predefined homogenous data collection protocol with a prospective study design, where possible.

### Emergency preparedness

The potential impact of using individualized group testing demonstrated herein with the AIP proposes a quadrupling of the testing capacity, and considerable savings within prevalence rates up to 50%, presumably with a proportional savings on plastic utensils, laboratory chemicals, power consumptions and CO_2_ emissions. To present an instance, as of May 2021, Denmark was performing ~ 200,000 rRT-PCR tests per day^37^, a capacity-increase only implemented due to prioritization over other analytic tasks. Quadrupling this amount means that the whole Danish population could have been tested in a 7- to 10-day period. The approach implemented in Denmark was a widespread enrolment of over-the-counter (OTC) rapid antigen test (RAT), which might be faster, but also diagnostic inaccurate, given its false-negativity risk prior the symptomatic-phase and prolonged false-positivity rates weeks after the infective period (communicability period)^38^. During the COVID-19 pandemic in Denmark, the RATs have been reported to produce 47% false negative results and 45% false positives^39^. Considering this, implementing Segtnan™ would have increased test capacity without compromising safety by utilizing the high diagnostic performance of rRT-PCR. Further, in times of economic constraints, of which many public healthcare systems around the world have been facing in the aftermath of COVID-19, it would be vitally important to save capital on diagnostic utensils and redistribute the available funding to the other diagnostic procedures that are in need. From a different perspective, Segtnan™ can be used to simply provide a sustainable increase in testing capacity, even in contexts without a severe economic restriction. As illustrated in Fig. 5b, even at a high prevalence rate of 15%, the testing capacity is doubled. Considering an extreme case with 50% disease prevalence, like an intensive care unit, the beneficial effects of AIP still maintain. The sustainable beneficial impacts also include the reduction in CO_2_ emissions and plastic use. This would be especially consequential with a global implementation of AIP and similar sustainable methods. Lastly, we showed that the system is feasibly applicable to the clinical day-to-day routines in various settings, which prepares us for the next medical emergencies.

### Automatization and Segtnan™

Adoption strategies for global implementation of the app is to increase the public knowledge about the technology and to partner with robotics collaborators to automize the manual procedures of pooling method. Dissemination explainers are made for ensuring the former, see https://www.segtnan.ai/segtnan-AIP, for an example. We are in collaboration with robotic teams and companies to connect Segtnan™ AIP on top of already-available medical sampling robotics (proof-of-concept made with ATRAS from T&O Labsystems), pipetting robots (architecture plug-in made with Flowbot One from Flowrobotics) and dedicated stand-alone robots (proof-of-concept AIP-Cobot made with Universal Robots). The details for the technology adoption that automatizes Segtnan™ AIP is beyond of the scope of this article.

## Conclusion

In this study, we have presented clinical validation for the use of an individualized AI-Integrated risk assessment method for pooling which was shown to improve testing efficacy. This makes it possible to implement an “individualized pooling” scheme in day-to-day clinical routine at patient wards, test centers and even emergency rooms. Moreover, we have validated the feasibility of integrating diagnostic data from other scientists directly into an equation for a data-driven diagnostically optimized pooling strategy. The results of the current study illustrated that exponential savings in usage of test kits and considerable reduction of economic costs were achieved at prevalence rates between 0.5 and 50%. Such an increase in testing capacity may prevent new lockdowns in case of future pandemics.

### Supplementary Information

Supplementary Information.

## Data Availability

The datasets gathered in this study are available from the corresponding author upon reasonable request. Given the confidentiality agreements with responsible institutions, the gathered datasets cannot be made publicly available. The mobile application, Segtnan™, is free to download for iOS and Android smart phones. Any patient/citizen can freely download the mobile application and obtain an optimal individualized pool size calculated via the Segtnan™.

## Abbreviations

AI: Artificial intelligence
COVID-19: Coronavirus disease 2019
SARS-CoV-2: Severe acute respiratory syndrome coronavirus 2
DE: Dorfman equation
AIP: AI-integrated risk assessment pooling
rRT-PCR: Real-time reverse transcription polymerase chain reaction
SE: Segtnan equation
ML: Machine learning
LR: Logistic regression
FNN: Feed-forward neural network
