# Supplementary material for: Clinical performance of AI-integrated risk assessment pooling reveals cost savings even at high prevalence of COVID-19
Source: Sci Rep. 2024 Apr 17;14:8853. doi: 10.1038/s41598-024-59068-6 (PMC11024139; doi:10.1038/s41598-024-59068-6)
Supplement: Supplementary file 1 — Supplementary Information. [file 41598_2024_59068_MOESM1_ESM.docx]

**Clinical performance of AI-integrated risk assessment pooling reveals cost savings even at high prevalence of COVID-19**

Farzin Kamari^1, +^, Esben Eller^2, +^, Mathias Emil Bøgebjerg^3^, Ignacio Martínez Capella^4^, Borja Arroyo Galende^5^, Tomas Korim^6^, Pernille Øland^7^, Martin Lysbjerg Borup^7^, Anja Rådberg Frederiksen^7^, Amir Ranjouriheravi^8^, Ahmed Faris Al-Jwadi^9^, Mostafa Mansour^10^, Sara Hansen^10^, Isabella Diethelm^10^, Marta Burek^10^, Federico Alvarez^5^, Anders Glent Buch^11^, Nima Mojtahedi^1^, Richard Röttger^3^, Eivind Antonsen Segtnan^12, 13*^

*Affiliations:*

^1^ Institute of Physiology, Department of Neurophysiology, Eberhard Karls University of Tübingen, Tübingen, Germany

^2^ Ellergy, Odense, Denmark.

^3^ Department of Mathematics and Computer Science, University of Southern Denmark, Odense, Denmark

^4^ Innovation Unit, IdISSC, Hospital Clínico San Carlos, Madrid, Spain

^5^ Grupo de Aplicación de Telecomunicaciones Visuales, Universidad Politécnica de Madrid, Madrid, Spain

^6^ Easyrobot, Bratislava, Slovakia

^7^ Hospital of Psychiatry, Odense, Denmark

^8^ Research Center for Translational Medicine (KUTTAM), Graduate School of Sciences and Engineering, Koç University, Istanbul, Turkey

^9^ School of Medicine at University of Southern Denmark, Odense, Denmark

^10^ SDU Health Informatics and Technology, Maersk Mc-Kinney Moller Institute, Faculty of Engineering University of Southern Denmark, Odense, Denmark

^11^ Department of Engineering, Maersk Mc-Kinney Moller Institute, Faculty of Engineering University of Southern Denmark, Odense, Denmark

^12^ Department of Neurosurgery, Odense University Hospital, Denmark

^13^ Synaptic ApS, Odense, Denmark

+ Kamari & Eller share first authorship and played complementary roles in this study.

*** Corresponding author**

Eivind Antonsen Segtnan, M.D., Ph.D.

Address: Skt Knuds Gade 20, 5000 Odense C, Denmark

E-mail: [Eivind@segtnan.ai](mailto:Eivind@segtnan.ai)

Telephone: 0045 30448498

**Supplementary Material**

1. **Danish and Spanish dataset demographics**

In the Danish dataset (Fig. 2, 1.2), there were slightly more females than males (53.2%, n = 916), and the mean ± SD of age was 44.7 ± 14.5. Most of the individuals (92.5%, n = 1590) did not report any symptoms nor contacts. Only a small proportion of the population (5.1%, n = 87) had a known contact while being asymptomatic. Overall, 2.4% of the individuals (n = 42) were symptomatic and the most common reported symptoms were sore throat (42.9%), cough (40.5%), headache (40.5%). From the symptomatic patients, all were stable at the time of testing, and no one had a severe clinical condition. 2 patients were on chronic corticosteroids and none of the patients were on any medications related to COVID-19, e.g., Remdesivir, Lopinavir/ritonavir, (Hydroxy)chloroquine, or Azithromycin.

In the Spanish dataset (Fig. 2, 1.3), the female gender dominated with 53.9% (n = 1181), and the mean ± SD of age was 61.2 ± 17.7 years. 47.2% of the individuals (n = 1035) did not report symptoms nor contacts, but 0.07% of the individuals (n = 150) did report a contact while being asymptomatic. 26.5% of the individuals (n = 580) were symptomatic but took no medications related to COVID-19 and 19.4% (n = 426), had symptoms and were on medications. 54.1% of the individuals (n = 1185) had a stable clinical condition, 20.2% (n = 442) reported a mild illness, 23.9% (n = 524) presented pneumonia (of which 60.9% was severe), 1.3% of the individuals (n = 28) had acute respiratory distress syndrome (ARDS), and less than 1% (n = 12) had sepsis or septic shock. Concerning symptoms, the most frequent ones were cough (62.3%), fever (56.8 %, of which 70.9% was low-grade fever, 17.2% has high-grade fever and 11.9% was fever of unknown temperature), dyspnea (30.8%) and headache (10.5%). In the case of medicated individuals, the most common medications were corticosteroids (48.1%), Lopinavir/ritonavir (39%) and Tocilizumab (13.3%).

1. **Machine learning and neural network training**

In the primary phase of the study, as a proof of concept, we only trained a CatBoost model^1^ (Fig. 2, left column) to show that an accuracy of 74% on the testing dataset (with a train\test split of 80\20) is possible with a small dataset size. The used dataset had 941 data entries, including 797 data entries from Literature study I, combined with 144 data entries with negative rRT-PCR test results randomly sampled from Danish clinical Study I (see Fig. 2). These two datasets were combined to equalize the imbalanced classes for a better ML training, i.e., to equalize the number of positive and negative results. Our aim of this pilot was to validate a questionnaire with relevant clinical questions, estimate the amount of economic savings and calculate the change in the testing capacity.

In the second phase of the study with datasets 1.2, 1.3 and 1.4 (in total 4,779 data entries), we used the PyCaret^2,3^ pipeline to fit the aggregated data on multiple well-known machine learning algorithms, to have a full comparison (Table S1). To find the best estimator of the pretest probability, we compared AdaBoost Classifier, CatBoost Classifier, Decision Tree Classifier, Extra Trees Classifier, Extreme Gradient Boosting, Gradient Boosting Classifier, K-Neighbors Classifier, Light Gradient Boosting Machine, Linear Discriminant Analysis, Logistic Regression, Naive Bayes, Quadratic Discriminant Analysis, Random Forest Classifier, Ridge Classifier, SVM-Linear Kernel. All models were optimized based on the accuracy metric.

**Table S1.** This table summarizes the results from different machine learning algorithms. Training time illustrates the required time for the training of the machine learning models (on CPU only) and the feed-forward neural network (on GPU) using the same dataset. SVM, support vector machine; AUC-ROC, area under the ROC curve; AUC-PR, area under the precision-recall curve.

| Model | Accuracy | AUC-ROC | AUC-PR | Recall | Precision | F1-score | Training Time (s) |
| --- | --- | --- | --- | --- | --- | --- | --- |
| Feed-forward neural network | 0.8138 | 0.8606 | 0.5878 | 0.7595 | 0.6168 | 0.6680 | 21.643 |
| CatBoost Classifier | 0.8014 | 0.8647 | 0.7210 | 0.7548 | 0.6090 | 0.6716 | 2.625 |
| Light Gradient Boosting Machine | 0.8009 | 0.8542 | 0.6985 | 0.7055 | 0.6197 | 0.6564 | 1.716 |
| Extreme Gradient Boosting | 0.7954 | 0.8417 | 0.6883 | 0.6871 | 0.6134 | 0.6444 | 1.618 |
| Random Forest Classifier | 0.7946 | 0.8313 | 0.6518 | 0.6713 | 0.6119 | 0.6367 | 1.432 |
| Extra Trees Classifier | 0.7938 | 0.7987 | 0.5948 | 0.6248 | 0.6189 | 0.6186 | 1.568 |
| Gradient Boosting Classifier | 0.7935 | 0.8680 | 0.7281 | 0.8151 | 0.5873 | 0.6805 | 1.472 |
| Ada Boost Classifier | 0.7925 | 0.8596 | 0.7032 | 0.8113 | 0.5862 | 0.6786 | 1.407 |
| Logistic Regression | 0.7901 | 0.8533 | 0.6616 | 0.8190 | 0.5808 | 0.6779 | 1.330 |
| Ridge Classifier | 0.7901 | - | - | 0.8315 | 0.5796 | 0.6814 | 1.333 |
| Linear Discriminant Analysis | 0.7893 | 0.8531 | 0.6598 | 0.8306 | 0.5786 | 0.6804 | 1.456 |
| SVM - Linear Kernel | 0.7891 | - | - | 0.8462 | 0.5766 | 0.6842 | 1.322 |
| Decision Tree Classifier | 0.7878 | 0.6915 | 0.5151 | 0.5777 | 0.6174 | 0.5927 | 1.312 |
| K-Neighbors Classifier | 0.7849 | 0.8228 | 0.5804 | 0.7351 | 0.5845 | 0.6479 | 1.316 |
| Quadratic Discriminant Analysis | 0.3089 | 0.5226 | 0.2776 | 0.9216 | 0.2649 | 0.4113 | 1.377 |
| Naive Bayes | 0.3079 | 0.8337 | 0.5831 | 0.9823 | 0.2819 | 0.4355 | 2.625 |

In Table S1, the metrics illustrates the validation metrics averaged from a 10-fold cross-validation. The formulations and pseudocodes for calculation of AUC-ROC and AUC-PR are given in refs^4,5^. Assuming TP, TN, FP, and FN to respectively denote true positives, true negatives, false positives and false negatives, the below metrics were calculated as formulized:

$$Accuracy = \frac{TP+TN}{TP+TN+FP+FN}$$

$$Recall = \frac{TP}{TP+FN}$$

$$Precision= \frac{TN}{TN+FP}$$

$$F1 score = \frac{2TP}{2TP+FP+FN}$$

1. **Feed-forward neural network training**

We trained a Keras^6^ multilayer feed-forward neural network with Adam optimizer and Bayesian tuner (50 epochs) on the aggregated dataset (1.1, 1.2 and 1.3). Interestingly, with an identical medium-sized datasets, we could obtain an accuracy of 81.38%, superior to that of CatBoost classifier; however, the training time of FNN was considerably higher (Table S1). In general, the FFN is outperforming machine learning classifiers when the training dataset size exceeded ~3000 data entries.

The training times for ML models were measured only with training on CPU, however, the training time of FNN was measured using both CPU and GPU. The hardware used were a processing unit of 2 x Intel Xeon CPU E5-2680 v3 @ 2.50GHz, 2501 MHz, 12 Cores, 24 Logical Processors, and a GPU of AORUS GeForce RTX™ 2080 XTREME 8G with 2944 CUDA cores. Regarding the inference speed, the elapsed time for both ML models and the FNN were less than a second per 1000 patients, and hence were negligible.

It is to be noted that the predicted pretest probabilities are further fed into the economic equations, so that the amount of savings is calculated. While it may be economically inferior not to miss any patient during the group testing, it is be more important to minimize the false negatives, since the false positives are discerned during the subsequent individual retests. Hence, the diagnostic accuracy, but not economic savings, of the smart pooling is increased with a discrimination threshold that favors recall.

1. **Class imbalance and multiple tests scenarios**

For clarity, we also performed the comparison between ML models in four scenarios (Table S2 and S3):

1. without excluding multiple tests per patients (in case they had during their disease) and with balanced classes.
2. with the exclusion of multiple tests per patients and with balanced classes.
3. without excluding multiple tests per patients and without fixing the imbalanced classes.
4. with the exclusion of multiple tests per patients and without fixing the imbalanced classes.

**Table S2.** Detailed information of the training and testing dataset sizes and class imbalance ratios in four analyzed scenarios. SMOTE, Synthetic Minority Over-sampling Technique; N: P, Negative to Positive.

| Comparison Scenarios | Primary dataset size | Training / Testing dataset size before SMOTE | Training / Testing dataset size after SMOTE | N: P ratio before SMOTE | N: P ratio after SMOTE |
| --- | --- | --- | --- | --- | --- |
| 1) including multiple tests + SMOTE | 4779 | 3823 / 956 | 5586 / 956 | 0.73: 0.27 | 0.5: 0.5 |
| 2) excluding multiple tests + SMOTE | 2728 | 2182 / 546 | 3536 / 546 | 0.81: 0.19 | 0.5: 0.5 |
| 3) including multiple tests, without SMOTE | 4779 | 3823 / 956 | - | 0.73: 0.27 | - |
| 4) excluding multiple tests, without SMOTE | 2728 | 2182 / 546 | - | 0.81: 0.19 | - |

**Table S3.** Comparison of the evaluation metrics of five top ML models, sorted by Accuracy, across four scenarios. AUC-ROC, area under the ROC curve; AUC-PR, area under the precision-recall curve.

| Scenarios | Top models | Accuracy | AUC-ROC | AUC-PR | Recall | Precision | F1-score |
| --- | --- | --- | --- | --- | --- | --- | --- |
| 1 | **CatBoost Classifier** | 0.8014 | 0.8647 | 0.7210 | 0.7548 | 0.6090 | 0.6716 |
|  | **Light Gradient Boosting Machine** | 0.8009 | 0.8542 | 0.6985 | 0.7055 | 0.6197 | 0.6564 |
|  | **Extreme Gradient Boosting** | 0.7954 | 0.8417 | 0.6883 | 0.6871 | 0.6134 | 0.6444 |
|  | **Random Forest Classifier** | 0.7946 | 0.8313 | 0.6518 | 0.6713 | 0.6119 | 0.6367 |
|  | **Extra Trees Classifier** | 0.7938 | 0.7987 | 0.5948 | 0.6248 | 0.6189 | 0.6186 |
| 2 | **Linear Discriminant Analysis** | 0.8887 | 0.9095 | 0.7359 | 0.8639 | 0.6624 | 0.7458 |
|  | **SVM - Linear Kernel** | 0.8882 | - | - | 0.8614 | 0.6618 | 0.7445 |
|  | **Ridge Classifier** | 0.8882 | - | - | 0.8639 | 0.6614 | 0.7451 |
|  | **Logistic Regression** | 0.8873 | 0.9145 | 0.7553 | 0.8617 | 0.6600 | 0.7431 |
|  | **Ada Boost Classifier** | 0.8850 | 0.9137 | 0.7680 | 0.8306 | 0.6596 | 0.7312 |
| 3 | **Gradient Boosting Classifier** | 0.8223 | 0.8714 | 0.7321 | 0.6403 | 0.6821 | 0.6574 |
|  | **Ada Boost Classifier** | 0.8171 | 0.8613 | 0.6993 | 0.654 | 0.6671 | 0.6565 |
|  | **Light Gradient Boosting Machine** | 0.8166 | 0.8615 | 0.7145 | 0.5937 | 0.6878 | 0.6329 |
|  | **CatBoost Classifier** | 0.8145 | 0.8685 | 0.7297 | 0.6113 | 0.6746 | 0.6382 |
|  | **Ridge Classifier** | 0.8087 | - | - | 0.6782 | 0.6403 | 0.6552 |
| 4 | **Gradient Boosting Classifier** | 0.8950 | 0.9191 | 0.7828 | 0.6839 | 0.7428 | 0.7046 |
|  | **CatBoost Classifier** | 0.8927 | 0.9199 | 0.7808 | 0.6833 | 0.7333 | 0.6996 |
|  | **Ridge Classifier** | 0.8923 | - | - | 0.7306 | 0.7139 | 0.7169 |
|  | **Logistic Regression** | 0.8914 | 0.9132 | 0.7515 | 0.7233 | 0.7155 | 0.7114 |
|  | **Ada Boost Classifier** | 0.889 | 0.9103 | 0.7780 | 0.7192 | 0.7155 | 0.7087 |

As shown in Table S3, higher accuracies are achieved in scenarios 2 and 4, which are with exclusion of patients who are having multiple tests. These results reveal that including multiple tests per patient brings a challenge for the ML models to learn the reality of the COVID-19 infection course. Although the patients with multiple tests do not entail an information leakage between the training and validation datasets, they provide a random-like noise which is inherently stemming from the fluctuations of the infection course. In other words, a patient that is positive in one day, can be negative the next day with similar symptoms. To obtain a generalized model which can predict the real-word cases, we intentionally included patients with multiple tests, so that the trained model can also learn the dynamics of the viral load (or say positive pretest probability) in the light of the days from the symptom onset variable and symptom fluctuations.

Interestingly, calculating savings and testing capacity indices, we found that although the accuracy increases in scenarios with multiple test exclusions, the economic savings is slightly reduced in these scenarios. This reduction is more significant for cost-neutral test-potential per 100 patients. One reason for the decrease in savings, despite the improved accuracy of the ML models, may stem from the fact there is less inference uncertainty (i.e., overstated pretest probabilities) in scenarios 2 and 4, and therefore, for many individuals, the proposed pool size turns out to be either individual testing or larger pool sizes. This may lead to practically unwise pooling and less savings. We believe training in scenario 1 is more robust for the real-world applications.

Additionally, as illustrated in Table S3, the use of SMOTE has not significantly changed the results (comparing scenario 1 with 3 and 2 with 4), but has created a bias toward the majority class, i.e., negative results. This is evident from an increase in precision and decrease in recall. Nonetheless, as mentioned earlier, one of our technical aims for Segtnan™ AIP smart pooling was to favor recall over precision, to be able to provide a method for wide-scale screenings.

1. **Pool size calculations**

The optimal pool size with the Dorfman method was calculated with the below equation^7,8^ (DE):

$$m=\left\lfloor\left. \frac{2}{\ln\left( 1-p \right)}\mathfrak{R}\left[ W(-\frac{1}{2}\sqrt{-\ln\left( 1-p \right)}) \right] \right\rceil\right.$$

where $m$ is the optimal pool size, $p$ is the positive pretest probability, $W$ is the $Lambert W$ function in mathematics, $\mathfrak{R}$ denotes the real part of a given complex number, and the ⌊⌉ is the mathematical notation for the convergent rounding function. It is to be noted that in Dorfman’s original article, it can be inferred that pooling shall not be performed after 30% of prevalence, as the total savings turn out to be negative. Despite this fact, for all prevalence rates, regardless of total cost function, we have calculated an optimal pool size based on his method considering a “forced pooling” condition. This was done to have a thorough comparison between the outcomes of our pooling method, Dorfman’s pools, and individual testing. As Dorfman is unable to propose any beneficial pooling in high prevalence rates after 30%, the method will propose the individual testing. The results for the individual testing have also been shown in the manuscript.

The Segtnan Equation (SE) was derived based on clinical data from other scientific studies on pooling. To derive the SE, we performed an exponential regression on the data from the reviewed articles in a recent study by Evangeline *et al.*^9^, in which the results of the most optimal pool size for any given prevalence is systematically gathered. The SE differs from the DE in that it is not based on finding the economic optimum. Instead, it is grounded from the results of the studies that have already successfully implemented group testing. That means the equation provides diagnostically- and clinically-sensible outcomes, rather than only optimizing the economic savings. Considering the same variables from the DE, the optimal pool size using the SE is obtained as:

$$m = \lfloor9.7069 exp(-0.058 p)\rfloor$$

where $\lfloor\rfloor$ denotes the round down notation and $exp$ is the exponential function. It is to be noted that the equation is used with simplified constants in the manuscript text. However, the regression result and performed calculations in the manuscript were as the equation above.

1. **Analysis of the economic savings**

The net monetary savings were calculated based on the regular fee of the regular rRT-PCR in the respective healthcare centers. Let $c$ be the cost for each test, which can be written as:

$$c=s+k+l$$

in which $s$ is the expense for sample extraction and human labor at the time of sampling, $k$ is the cost for the required chemicals, equipment depreciation and human labor in the laboratory, and $l$ is the cost for transportation and logistics. And let $n$ be the total number of people to be tested, and $g$ the number of groups, i.e., pools, that has been proposed by the AI, then in the first round of pooling, the first-round expenses ($e_{1}$) can be formulated as:

$$e_{1}=n(s+l)+gw+gk$$

where $w$ is the cost for the extra workload needed for the logistics and handling of pools. In automated pooling systems (see discussions), $w$ can be negligible and shall be substituted by the automating robotics hardware depreciation/maintenance costs. Nonetheless, in the conventional pooling labs where the human labor is responsible for the pooling logistics, $w$ can be assumed to be similar to $l$ (i.e., $w\approx l$) and hence the above equation can be simplified as:

$$e_{1}=n(s+l)+g(k+l)$$

Next, from those groups that are turn out to be positive in the first round, assuming $z$ to be the number of groups each with $m_{z}$ members, all individuals are tested individually in the second round, leading to the second-round expenses ($e_{2}$) to be:

$$e_{2}=\sum_{i=1}^{z} m_{i} k$$

Hence, after the second round, all the positive individuals will be identified. The total savings ($t$) can then be calculated as:

$$t=nc-(n(s+l)+g(k+l)+\sum_{i=1}^{z} m_{i} k)$$

The above equation was used to calculate the economic savings in this manuscript. Calculating the total savings $t$, the saving per each 100 patients ($t_{100}$), and the pooled testing cost per each 100 patients ($q_{100}$) can be written as:

$$t_{100}=\frac{t}{n}\times100$$

$$q_{100}=(1-\frac{t}{n})\times100$$

We gained the information for variables $c$*,* $s$*,* $k$ and $l$ based on informal interviews with personnel at two different laboratories in Spain and Norway, where $c=75$ €, $k=61.34$ €, $s=9.08$ €, $l=4.58$ €. It was not possible to obtain official written information from authorities regarding the rRT-PCR costs.

1. **Testing capacity and other implications**

Considering the same variables from the above section, the change in the testing capacity (δ) can be calculated as:

$$\delta=\frac{n}{g+\sum_{i=1}^{z} m_{i}}-1$$

And $\delta\times100$ gives the amount of change in the testing capacity in percentage. The change in the testing capacity and the reduction in the number of the required tests will have a direct impact on the utilization of plastic accessories, reagents, and CO_2_ emissions produced in relation to the testing procedure.

Furthermore, an index of cost-neutral test-potential per 100 patients tests ($\psi_{100}$) can be defined, based on the previously derived amount of savings and the amount of pooled testing cost per 100 patients. On a public health level, $\psi_{100}$ is eminently critical, because it directly shows the potential impact of pooling after its implementation in the society. $\psi_{100}$ can be formulated as:

$$\psi_{100}=\frac{t_{100}}{q_{100}}$$

Moreover, concerning the other implications, an exact carbon footprint analysis is out of the scope of our study; nonetheless, herein we present estimations based on the change in the usage of plasticware. The amount of savings in plastic ($\Pi$; in percent) is fairly proportionate to the number of the utilized diagnostic assays. Therefore, $\Pi$ can be formulized as the ratio of unused laboratory plasticware to total plasticware used during individual testing:

$$\Pi=\frac{n - (g+\sum_{i=1}^{z} m_{i})}{n}\times100$$

Accordingly, the amount of change in CO_2_ emissions can be further estimated based on the available emission conversion charts for plastic^10^, and the used plastic per each tube and cap, as well as emissions per used chemicals and energy^11,12^ per sample. Note that the potential reduction in CO_2_ emissions related to the logistics optimization and transport reduction due to the online reporting system has been left uninvestigated in the current work. The emission conversion charts provided an estimate of 36.6 g CO_2_ emission per one rRT-PCR test. Hence, a reduction in test numbers by 50% would lead to a 1830 g reduction in CO_2_ emissions per 100 samples tested. Consumption of other resources would also directly be linked to the number of performed tests and would be reduced when Segtnan™ AIP is applied.

**References**

1 Prokhorenkova, L., Gusev, G., Vorobev, A., Dorogush, A. V. & Gulin, A. CatBoost: unbiased boosting with categorical features. *Advances in neural information processing systems* **31** (2018).

2 Ali, M. *PyCaret: An open source, low-code machine learning library in Python*, <<https://www.pycaret.org>> (2020).

3 Pedregosa, F. *et al.* Scikit-learn: Machine Learning in Python. *Journal of Machine Learning Research* **12**, 2825-2830 (2011).

4 Fawcett, T. An introduction to ROC analysis. *Pattern Recognition Letters* **27**, 861-874, doi:10.1016/j.patrec.2005.10.010 (2006).

5 Keilwagen, J., Grosse, I. & Grau, J. Area under precision-recall curves for weighted and unweighted data. *PloS one* **9**, e92209 (2014).

6 Chollet, F. & Others. Keras. (2015).

7 Dorfman, R. The Detection of Defective Members of Large Populations. *The Annals of Mathematical Statistics* **14**, 436-440, doi:10.1214/aoms/1177731363 (1943).

8 Batson, J., Bottman, N., Cooper, Y. & Janda, F. A comparison of group testing architectures for COVID-19 testing. *arXiv preprint arXiv:2005.03051* (2020).

9 Daniel, E. A. *et al.* Pooled Testing Strategies for SARS-CoV-2 diagnosis: A comprehensive review. *Diagnostic Microbiology and Infectious Disease* **101**, 115432 (2021).

10 The National, A. *Greenhouse gas reporting: conversion factors 2020 - GOV.UK*, <<https://www.gov.uk/government/publications/greenhouse-gas-reporting-conversion-factors-2020>> (2020).

11 Winnipeg.ca. *Emission factors in kg CO2-equivalent per unit*, <<https://www.winnipeg.ca/finance/findata/matmgt/documents/2012/682-2012/682-2012_Appendix_H-WSTP_South_End_Plant_Process_Selection_Report/Appendix> 7.pdf> (2012).

12 IEA. *Global Energy & CO2 Status Report 2019*, <<https://www.iea.org/reports/global-energy-co2-status-report-2019>> (2019).
